# Research on the Long-Term Governance Mechanism of Urban and Rural Living Environment Based on the Ordered Logistic-ISM Model in the Perspective of Sustainable Development

**DOI:** 10.3390/ijerph191912848

**Published:** 2022-10-07

**Authors:** Yufeng Li, Ziwei Huang, Yonghang Li, Pu Xu

**Affiliations:** 1 School of Economics and Management, Shanghai Ocean University, Shanghai 201306, China; 2 Comprehensive Development Office, Shanghai Pudong New Area Agriculture and Rural Committee, Shanghai 201304, China

**Keywords:** urban and rural residential environment, governance effectiveness, orderly logistic-ISM model, long-term management

## Abstract

(1) Background: Even though the quality of life in urban and rural areas is better than in traditional rural villages, it is hard to keep up good governance over the long term. Exploring the limitations of villagers’ participation in the long-term management of urban rural habitat is exemplary from the perspective of sustainable development in order to improve rural habitat, promote sustainable economic and environmental development, and accomplish rural revitalization. (2) Methods: Long-term management indicators were proposed to provide a technique for evaluating the governance efficacy of urban and rural living environments. Based on the survey data obtained in Shanghai, the ordered logistic-ISM model was used to examine the influencing aspects of urban and rural living environment governance efficacy. (3) Results: In addition to environmental improvement conditions and other factors, long-term management in the village has a significant favorable impact on the evaluation of the governance effect on the living environment in urban and rural locations. (4) Conclusions: Long-term management directly supports villager engagement in environmental development and village infrastructure improvement and has a significant positive impact on the governance of urban and rural living environments. Improvements in infrastructure immediately benefit the administration of urban and rural living situations. Creating a long-term management structure, supporting cooperative governance, optimizing corrective actions, coordinating investments, and advancing ecological and economic sustainability are just a few of the policy improvements suggested.

## 1. Introduction

The major purpose of China’s full execution of the rural revitalization policy is to improve rural living circumstances. Urban and rural areas are the forerunners of strategy implementation in order to promote the balanced development of urban and rural areas in the new era. The No. 1 Central Document of China in 2022 also advises that the five-year action to improve the rural living environment be continuously implemented in order to consolidate present governance gains. However, due to the substantial financial disparity and the lack of long-term solutions [1,2], rural areas in China are usually “unbalanced, unclean, and messy” [3,4]; have insufficient villagers’ engagement [5]; and lack cross-sector cooperation [6]. Because of the expansion of industries in cities, urban rural areas have greater habitat quality and treatment than traditional rural areas. Maintaining and sustaining treatment, on the other hand, remains a barrier, creating a conundrum for long-term economic and environmental development in urban and rural areas.

Shanghai is one of the best examples of an international metropolis. How to accomplish and maintain sustainable development of rural economies and habitats is a necessary practical issue for Shanghai’s development as a global city. Traditional government-led infrastructure development and living environment optimization have been the primary focuses of rural living environment governance, but as urban and rural economic foundations have strengthened, the emphasis on villagers’ participation and long-term governance has shifted. The problem of insufficient efficacy in urban and rural human settlement governance can be remedied by determining how to effectively stimulate villagers’ participation in environmental governance and attain long-term and sustainable human settlement governance. Tourism, leisure, and environmental preservation are also becoming increasingly essential in the countryside as it evolves into a whole system of “production, living, and ecology” [7,8]. Shanghai also proposes establishing a “Beautiful Home” initiative to improve the rural environment. It will encourage farmers to live in relatively dense and orderly communities by building demonstration villages, implementing laws such as “creating beautiful courtyards” and “waste classification”, as well as optimizing the layout of rural places and promoting rural Shanghai. Society has paid more attention to the human environment in traditional rural areas, focusing on infrastructure construction and the optimization of the living environment while paying less attention to the sustainability of urban and rural habitats and efficacy of governance. As a result, while focusing on modern urban rural development, it is critical to clarify the problems in urban rural habitat governance from the perspective of villagers, include long-term governance indicators to measure the sustainability of urban rural habitat governance, and analyze the constraints of urban rural habitat and logical relationship between the factors, which will serve as a model for achieving sustainable economic and environmental development.

## 2. Literature Review

### 2.1. Concept of Urban and Rural Living Environment

Traditional rural areas are separate from urban rural areas. It is a new way of life in the countryside that has evolved with the growth of modern cities. It is regarded as a modern rural area in urbanized areas and surrounding areas that is reliant on the superior resources of large cities and closely serves the cities [9], implying that the urban and rural living environment not only has the general characteristics of the traditional rural environment but also has the characteristics of high urbanization and obvious economic advantages and carries the radiation of urban production and living functions. The term "urban and rural living environment" in this essay refers to a comprehensive system that preserves the ecological benefits of rural areas while also improving modern agriculture, carrying out various sophisticated jobs, and supporting sustainable development. Rural modernization has naturally improved living circumstances in both urban and rural communities. The development of both urban and rural areas is critical to the process of urbanization [10]. Cities can improve their overall competitiveness by focusing on the coordinated growth of the economy, ecology, and society in urban and rural areas.

### 2.2. Literature Related to Rural Habitat Evaluation Index System

Improving rural living circumstances can considerably promote rural revitalization [11]. The ecological value of rural areas significantly contributes to the national economic value and has a direct or indirect positive impact on people’s well-being [12]. The vast bulk of relevant research is undertaken in traditional rural areas. Researchers have created a framework for evaluating the rural living environment from the perspectives of enterprises and the government in terms of physical, ecological, and humanistic components [13,14,15,16,17,18]. Traditional culture, infrastructure, and living conditions, according to them, are the primary effects on the traditional rural living environment [19,20]. Sustainability of governance and community engagement have a greater impact on the management of urban and rural human settlements [21]. Some academics have remarked that China’s rural environment continues to suffer from unequal regional development [14], as seen by the high level of development in the eastern coastal districts and the backwardness in the southwest region [15,18], where there is a low level of environmental preservation and financial commitment is lacking [17]. The improvement of living circumstances in urban and rural communities is a deliberate, long-term effort [22]. The government promotes strong collaboration between enterprises and other social organizations in order to successfully increase the standard of urban and rural environmental governance and develop economic and environmental sustainability [5,23]. It also teaches villagers how to actively participate in environmental governance. Long-term management is a modern management approach that aims to achieve long-term management effectiveness by beginning with the object of governance, concept of governance, and main body of governance and shifting the traditional management concept from a unitary governance model dominated by the government to a pluralistic governance model in which various social bodies collaborate. The goal of the long-term management of urban rural habitat environment, as defined in this paper, is to achieve sustainable urban rural habitat environment management through a model that includes village participation, government leadership, long-term effective governance outcomes, and beneficial governance mechanism development.

### 2.3. Literature Summary

Academics have focused their emphasis on traditional rural areas, whereas urban and rural areas have received less attention. Economic growth and rural modernization are the key issues of current research in both urban and rural areas, but villagers’ participation and long-term governance are given little attention [24]. Because of the disparities in economic development levels across the country, there are recurring inequities in the management of the rural living environment. Traditional rural living environment governance prioritizes infrastructure construction and living environment enhancement. More focus should be placed on villagers’ engagement and long-term governance sustainability in the governance of urban and rural living settings. There is, however, little relevant research on the contrast between the focus of traditional rural and urban rural living environment governance, and the staged distinctions in governance in different regions are less involved. Based on these findings, this study developed an assessment index system for the efficiency of governance in the urban and rural living environments, as well as long-term governance indicators to quantify the sustainability of governance in the urban and rural living environments. The logistic-ISM model explores the long-term controlling mechanism of urban and rural human settlements, its limiting restrictions, and the correlation and hierarchical structure of each aspect from the perspective of the villagers. Shanghai, as a representative city, is expected to effectively improve long-term management of urban and rural living conditions and serve as a model for the modernization of both traditional urban and urbanized rural areas.

## 3. Theoretical Hypothesis

### 3.1. Long-Term Management, Environmental Improvement, and Effect of Urban and Rural Living Environment Governance

The theory of polycentric governance is one of the components of public management philosophy. Academics who believe that single-center control of the government may be ineffective suggest a polycentric system that restricts and supervises each other in the process of collaboration [25,26]. To compensate for the lack of governance efficiency in the single-center government system, the multicenter governance theory constructs a multicenter governance model within the three-dimensional framework of government, market, and society [27]. A common example of a public good is the natural environment. There are many circumstances in life where one can get by without paying. The features of a strong public good can also be found in rural living conditions [28]. The rising needs of urban and rural environmental development cannot be satisfied if unitary government control is maintained. Villagers engage with the government and enterprises on their own initiative to solve the growing requirements of rural residents for a better life, which can boost the effectiveness of rural living environment governance. Long-term rural environmental management is a thoughtful and rigorous endeavor. Another goal of the rural regeneration method is the long-term management and construction of rural human settlements. To practice long-term management, governments at all levels must work together with communities to improve the environment. As a result, long-term management of village participation will improve rural living environment governance, provide an inherently motivating element for villagers’ environmental protection, and promote both urban and rural sustainable development. This supports hypothesis 1 proposed in this paper.

**H1:**  *The governance of urban and rural living environments benefits from the long-term management of villagers’ participation.*

The complexity and diversity of the public’s wants are increasing. Outsourcing and decentralization of governmental operations have resulted in the concept of collaborative governance [29]. Collaborative governance manages the coordination role of the government through intermediary entities and soft influence models. Coordination is a type of polycentric governance that is often used in environmental governance, with the underlying idea that cooperation promotes confidence and improves the efficacy of governance activities [28]. Based on the framework of “attitude–situation–behavior”, some researchers have proposed that attitude factors and external conditions interact to influence behavior [30], and they contend that villagers’ environmental responsibility behavior is also a result of the interaction of internal environmental attitudes and external environmental factors. Today’s government-led governance is incapable of dealing with the complex state of rural environmental rehabilitation. The government’s support of local participation in environmental rehabilitation is a new trend in joint environmental control between urban and rural areas. Increasing villagers’ pride in their communities may improve their enthusiasm for environmental preservation and encourage their active participation in local environmental restoration. Based on this, hypothesis 2 was developed in this study.

**H2:**  *The coordinated environmental improvement by the villagers has a favorable effect on the enhancement of the living conditions in both urban and rural areas.*

### 3.2. Infrastructure, Ecological Environment, and Urban and Rural Living Environment Governance Effectiveness

Regional sustainable development theory relates to how to address the needs of specific regions through comprehensive and equitable development while also taking into account subsequent development and not undermining the needs of other regions. Studying sustainable development systems, which are composed of three interconnected subsystems that are dependent on the social, economic, and environmental sectors, is a dynamic process [31]. Some scholars refer to the sustainable development system and divide rural human settlements into soft and hard environments based on the scientific concept of human settlements [11]. Culture, autonomy, coordination, and so on are examples of the soft environment [32], whereas infrastructure, the ecological environment, and so on are examples of the hard environment [33]. The living environment will benefit from improved housing conditions, infrastructure, climate, garbage sanitation, and other things. A complete infrastructure in the village, including access to water, power, education, and medical care, can substantially improve the convenience and enjoyment of the people. The improvement of the ecological environment, such as garbage sanitation, is a critical aspect in improving the appearance of the village and developing both urban and rural environmental governance. As a result, hypotheses 3 and 4 were proposed in this work.

**H3:**  *The governance of urban and rural living environments is positively impacted by the state of the infrastructure.*

**H4:**  *The governance of urban and rural human settlements is positively impacted by the state of the natural environment.*

### 3.3. Intrinsic Relationship between Factors

The autonomy of villagers to participate in village affairs improves the level of modernization of government organization and management, alleviates the common issue of rural residents’ lagging self-governance, and promotes the formation of a long-term governance mechanism for the urban and rural environments. To achieve long-term management, it is critical to begin with environmental improvement. Environmental improvement can have a direct impact on both the infrastructure and natural environment. The infrastructure and natural environment are direct effects of better urban and rural living environments’ governance. The hard living environment, which directly impacts the appearance of the village and enhances people’s standard of life, comprises public facilities such as water, power, and transportation, as well as ecological conditions such as garbage and sanitation [33]. The soft environment of human settlements, in particular, spiritual beliefs and thought patterns found in culture and folklore, eventually influences the villagers’ feelings and behavioral orientation toward the community. The infrastructure and natural setting are hard environmental components in the village, and there is some interaction with soft environmental aspects such as cultural ambiance and villager autonomy [11]. The long-term management method may have an impact on villagers’ participation in environmental remediation. The residents’ environmental restoration initiatives may have an influence on the infrastructure and natural environment. The mutual coupling of various variables promotes the optimal development of urban and rural living environment governance. Based on this, this essay proposed hypothesis 5 and built a theory analysis framework.

**H5:**  *The internal logical relationship among the factors is long-term management in the village→environmental remediation conditions→ecological environment conditions and infrastructure conditions.*

**Figure 1:**
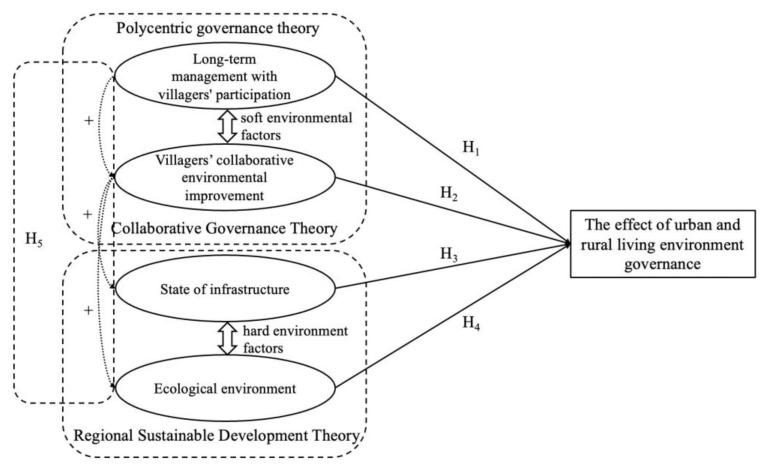
Theoretical analysis framework.

The theoretical analysis framework is shown in Figure 1.

## 4. Materials and Methods

### 4.1. Data Sources

**Figure 2:**
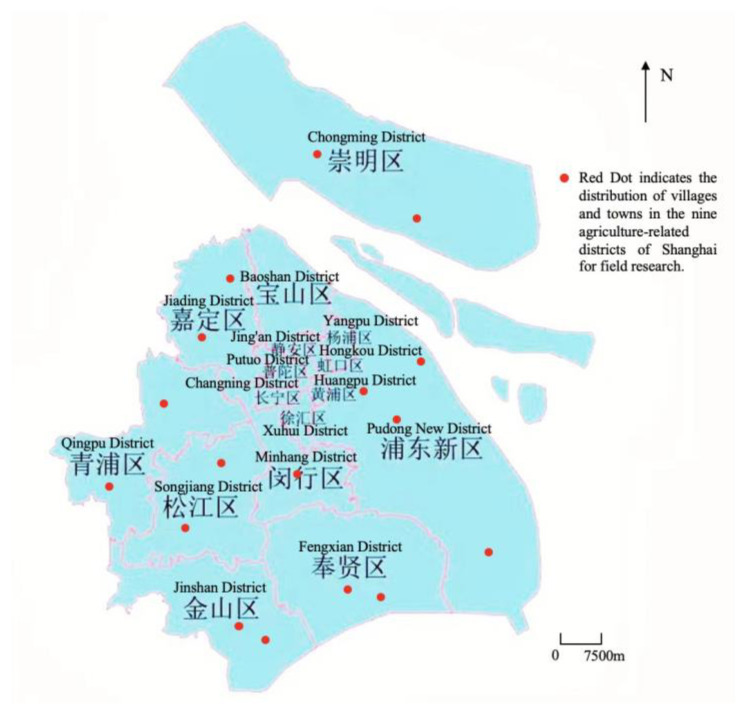
Map of survey sample sites.

This study’s findings are based on a survey conducted in 2021 in nine Shanghai areas with a major agriculture industry. A random selection technique was employed to deliver questionnaires to villages in rural areas in numerous Shanghai districts. The questionnaires’ main topics are the improvement and evaluation of the rural living environment. Prior to data analysis, sample screening was performed since some village survey index data were missing, irregular, and so on. There were 280 valid questionnaires received, with a valid sampling percentage of 98.2%. Figure 2 shows a map of the survey sample sites. The survey included eight agriculture-related districts, covering 88.89%, and the villages were chosen from different categories such as model and nonmodel villages for rural revitalization, as well as built-up and nonbuilt-up areas, covering a diverse range of villages with strong representation. The specific questionnaire design is in Appendix A.

### 4.2. Variable Selection and Sample Characteristics

#### 4.2.1. Explained Variable

According to certain academics who cite current studies, the advancement of human settlements benefits peasants. It is believed that the level of rural environmental governance can be more precisely gauged from the perspective of villagers’ subjective emotions [2,17,26] as the last manifestation of the effectiveness of rural environmental restoration. This paper drew on earlier research and selected the villagers’ assessment of the governance of the rural living environment to examine the efficacy of the governance of the urban and rural living environments. “High”, “normal”, “low”, and “very low” are each allocated 5 points.

#### 4.2.2. Core Explanatory Variables

The index system employed in this study was developed by consulting earlier research, combining China’s 19th National Rural Revitalization Strategy with relevant Shanghai policy papers, and was based on three elements of urban–rural field research. According to research on the living, ecological, and humanistic environment evaluation index systems of the rural habitat environment, improving the rural habitat environment focuses on addressing issues such as challenging toilets, a dirty environment, a rundown appearance of the village, outdated infrastructure and public services, and inadequate endogenous system construction [2,3,13,14,15,16,17,18]. The 19th National Congress of China issued general criteria for the rural rejuvenation policy, which included topics such as ecological sustainability, rural-style civilization, and effective government. Ecological livability entails improving the appearance and infrastructure of the village, such as water, electricity, and roads; rural-style civilization entails the development of the village’s culture, education, and health care; and effective governance emphasizes the need to establish and implement a modern rural social governance system with government leadership, social coordination, and public participation. In response to this requirement, Shanghai has developed the “2022 Shanghai Rural Habitat Optimization Project Task List”, which covers a wide range of topics such as village appearance, addressing infrastructure deficiencies such as the lack of public restrooms, improving rubbish management, improving road quality, improving the convenience of using restrooms, and establishing a reliable long-term management and care mechanism. These data form the basis for the development of evaluation indicators for four categories in this paper: long-term administration, environmental enhancement, infrastructure, and ecological environment. The secondary indicators are developed in cooperation with village interviews and cover the use of toilets, village appearance, river water clarity, road building, and other common concerns of the villagers’ habitat management points. Finally, they determine villagers’ participation as well as the other 25 indicators that will be used to construct the urban–rural habitat environment management effectiveness evaluation index system. The specific indicators were selected on the basis shown in Table 1. In this study, factor analysis was performed to ensure that the index system was rationally created. The reliability and validity test of the data using SPSS showed that Cronbach’s alpha was 0.941, the Kaiser–Meyer–Olkin value was 0.941, and the Bartlett *p*-value was 0.000, which was significant and indicated that the scale was suitable for factor analysis. The meaning of the indicators is presented in Table 2. The factor loading coefficients after rotation are shown in Table 3.

The extraction produced four variables, with a cumulative variance contribution rate of 67.507%. The classification of each index was consistent with predictions, indicating that the index system was effectively constructed. According to the variable nomenclature established by researchers [11,12], this work identified the four retrieved factors as LTM (long-term management in the village), ERS (environmental remediation), IS (infrastructure), and EE (ecological environment) and as the key explanatory variables.

#### 4.2.3. Control Variables

Villagers’ perceptions of the success of rural habitat management are also influenced by factors such as family wealth, educational attainment, and age. For example, the more educated the peasants, the better their appraisal of the rural environment [28]. The older generation of villagers has lived in the village for a long time, is more accustomed to the village’s original environmental appearance, and anticipates that their children will not live there. As a result, they lack the autonomy to actively participate in village environmental management. Throughout the year, the younger generation of villagers spends the majority of their time working outside the town [5]. Villagers’ demands on the environment are more likely to be higher [5,18]. When village committee and villager interviews were combined, it was discovered that village committees had higher opinions of the urban rural habitat environment, villagers who identified with their villages as models had stronger feelings about them, and villagers in catchment areas had better village planning and construction and were generally more complimentary of them. As a result of this, this study combined Shanghai’s characteristics and customs with relevant literature on peasants’ satisfaction with and evaluation of human settlements [17,18], and it selected seven indicators, including sex as a control variable. The sex, village cadre, village revitalization demonstration, and collective construction mean values were compared and assessed. The governance efficacy of rural revitalization demonstration villages and villagers in collective construction areas was much higher than that of nonvillage cadre status, nonrural revitalization demonstration villages, and noncollective construction area villagers. This could be because village cadres are more likely to develop cultural self-confidence, are more active in environmental conservation on an as-needed basis, and have a sense of responsibility for the village’s environment. They naturally have a more favorable impression of the village’s living conditions. The environmental design of the rural regeneration demonstration villages is more rigorous and detailed, and the people are frequently awarded higher evaluations. Villagers in the collective construction region live close to the city; housing is planned and built in a coordinated manner; housing conditions are good; nonwage income, such as housing rental, is high; life is convenient; and the appraisal of the governance effect is higher. The control variable description and mean comparison results are shown in Table 4.

### 4.3. Ordinal Logistic Regression Model

Multicollinearity was not an issue in this study because the collinearity diagnosis was completed prior to the regression, and each factor’s variance inflation factor value was less than 10. A multivariate ordinal logistic regression analysis was selected, then a backward removal on the data was performed before running the regression analysis again. Model 1 includes all independent factors that may have an effect on the dependent variable, and variables with a significant level of more than 10% are discarded based on the test findings in model 2. Models 1 and 2 have the same expressions as Equation (1), where $P_{i}$ denotes the likelihood that the villager $X_{i}$ will have an impact on the governance of the rural settlement environment, $\alpha_{i}$ denotes the intercept, $\beta_{i}$ denotes the regression coefficient of the corresponding explanatory variable, and $X_{i}$ denotes the ith villager.

(1) $$ln(\frac{P_{i}}{1-P_{i}})=\alpha_{i}+\beta_{i}\cdot X_{i}$$

### 4.4. ISM Model

Ordinal logistic regression cannot reveal the deep-level relationship between urban and rural human settlements, environmental governance, and these important influencing factors. The linkage and hierarchical structure of the aforementioned components will thus be investigated in the following utilizing the ISM model. S_0_, S_1_, S_2_, S_3_, and S_4_ were used to represent the evaluation of urban and rural living environment governance effectiveness, LTM, ERS, IS, and EE, respectively, based on the findings of ordered logistic regression. Through investigation, debate, and engagement with relevant specialists, the logical relationship between the four aspects defining the effectiveness of rural living environment governance was discovered. “N” signifies the row factor’s direct or indirect effect on the column factor, “U” denotes the column factor’s direct or indirect influence on the row factor, and “0” denotes the absence of an influence relationship [34].

The transformation of the logical relationship diagram yields the adjacency matrix *R*. A value of “1” indicates influence, whereas a value of “0” indicates no influence. The factor with the greatest influence is chosen as the influence for the factors that have mutual influence. The reachable matrix *M* is generated using Formula (2), and, finally, the hierarchical structure between the factors $L_{i}$ is determined by Formulas (3) and (4).

(2) $$M={\left(R+I\right)}^{\lambda +1}={\left(R+I\right)}^{\lambda }\neq {\left(R+I\right)}^{\lambda -1}\neq \cdots \neq {\left(R+I\right)}^{2}\neq \left(R+I\right)$$

In the formula 2≤λ≤n, “*I*” is the identity matrix, and the matrix exponentiation operation uses the Boolean algorithm.

(3) $$L_{i}=\left\{S_{i}/P\left(S_{i}\right)\cap Q\left(S_{i}\right)=P\left(S_{i}\right),i=0,1,2,3,\cdots ,n\right\}$$

(4) $$P\left(S_{i}\right)=\left\{S_{i}|r_{ij}=1\right\},Q\left(S_{i}\right)=\left\{S_{i}|r_{ji}=1\right\}$$

$L_{i}$ represents the set of ith layer factors, $r_{ij}$ and $r_{ji}$ represent the elements in the reachability matrix *M*, $P\left(S_{i}\right)$ represents the reachable set, and $Q\left(S_{i}\right)$ represents the antecedent set. The reachability matrix was divided into levels, and an explanatory structural model was built based on the computation results (omitted). The factors were divided into the highest-level factor L_1_ = {S_0_}, and then the rows and columns corresponding to the factor L_1_ were deleted from the reachability matrix. Equation (3) was calculated to obtain the second layer L_2_ = {S_3_,S_4_} and so on to obtain L_3_ = {S_2_} and L_4_ = {S_1_}. The rows and columns of the reachable matrix were rearranged in accordance with the result of factor-level division to generate the hierarchical structure matrix *B*.

## 5. Results and Discussion

The regression results of the ordered logistic model are shown in Table 5. At the 1% level, LTM, ERS, IS, and EE greatly increase villagers’ views of the efficiency of government in urban and rural living settings. The impact of the six villagers’ characteristics on the efficacy evaluation of urban and rural living environment governance is not immediately obvious. This could be because, as people’s thinking develops, sex and age distinctions become less essential, and they begin to hold more objective and rational viewpoints. Sex and age had little influence on villagers’ assessments in Shanghai’s more developed rural areas. Because of the government’s regular advertising of environmental governance legislation and the steady increase in villagers’ understanding of environmental preservation, villagers may downplay the importance of their educational background and unique village characteristics on the efficiency of governance. Many unclear factors, such as the market and climate, will have an impact on family income, and villagers will find it difficult to evaluate the contribution of income to the efficacy of rural living environment governance. This could be because village cadres have higher institutional and interpersonal trust, which causes villagers to pay more attention to and recognize policies, as well as to feel a greater sense of responsibility for the environment, regardless of whether they are significant at the 10% significance level or not. However, because Shanghai’s rural environment is superior to that of other regions, the two are often identically scored.

The villagers’ identities and localities may have different sentiments regarding the rural living environment, and there may be conflicts in evaluation due to data and variable restrictions. Another concern is bias in sample selection. As a result, grouping regression is employed to perform a robustness test in this research. The results of the heterogeneity analysis demonstrate that LTM, ERS, IS, and EE have a significant and consistent influence on villagers’ views of the effectiveness of governance in both urban and rural living contexts after grouping. The results have been proven to be reliable. The robustness test results are shown in Table 6.

**Figure 3:**
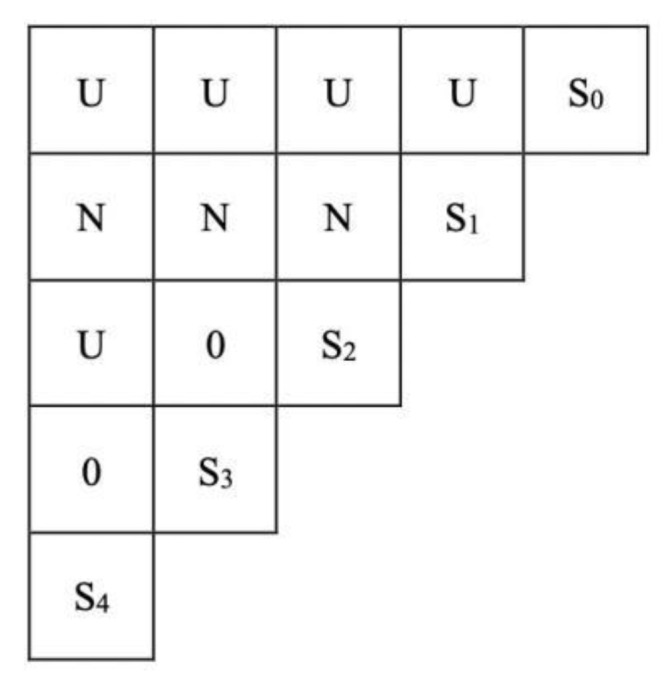
Logical relationship between factors. S_0_, S_1_, S_2_, S_3_, and S_4_ represents the evaluation of urban and rural living environment governance effectiveness, LTM, ERS, IS, and EE, respectively. “N” signifies the row factor’s direct or indirect effect on the column factor, “U” denotes the column factor’s direct or indirect influence on the row factor, and “0” denotes the absence of an influence relationship.

According to the expert scores, the logical relationship between the four aspects is shown in Figure 3. According to the findings, the four main factors that are directly or indirectly impacted by the evaluation of the efficacy of urban rural habitat management are LTM, ERS, IS, and EE. LTM has an impact on ERS, IS, and EE, either directly or indirectly. ERS also has an impact on EE, either directly or indirectly, but there is no relationship between ERS and IS, and IS and EE.

Based on calculations, the hierarchical structure matrix *B* is shown in Formula (5).

(5) $$B=\begin{array}{c} S_{0}\\ S_{3}\\ S_{4}\\ S_{2}\\ S_{1} \end{array}\begin{array}{c} S_{0}\;S_{3}\;S_{4}\;S_{2}\;S_{1} \end{array}$$

According to an assessment of the underlying core causes, long-term management in the village has a significant positive impact on the governance of urban and rural living environments. The validity of hypothesis 1 demonstrates that the greater the villagers’ willingness to participate, the better the long-term management mechanism will be constructed, and the governance of both urban and rural living environments will be improved. Villagers are direct participants in the rural living environment, and the start and continuation of environmental cleanup activities are closely related to government leadership. Independent participation in village affairs can promote a sense of ownership, improve the authority of co-inherent governance, and contribute to the development of long-term management systems. The many effects of the government, other parties, and the people all have an impact on how well the village manages its environment.

According to the examination of indirect factors at the middle level, the condition of environmental remediation has a significant positive impact on the effect of urban and rural living environment governance. The second hypothesis is confirmed, demonstrating that the better the village’s environmental remediation situation, the higher the quality of life in both urban and rural areas; and the more effective the village government is, the more willing the villagers are to participate in environmental remediation. The government can considerably address the village’s issues of uneven housing and underutilized land resources by encouraging residents to live together in a concentrated manner. It is impossible to divorce government funding from the improvement and management of the village’s roads, streams, and other ecosystems. The villagers’ voluntary care, protection, and engagement in their participation are far more important. Environmental remediation can successfully reduce the cost of government environmental governance communication while increasing governance efficiency.

According to an assessment of direct elements at the surface level, both the biological environment and infrastructural circumstances have a considerable positive influence on the administration of urban and rural human settlements. The validity of hypotheses 3 and 4 demonstrates that the influence of environmental management grows in proportion to the quality of the village’s ecological environment. The government’s consistent and dependable financial support can assure the long-term development and upkeep of the village’s infrastructure and natural environment. The natural environment can represent the village’s environment in the same manner as infrastructure reflects the constancy of government spending, which, in turn, directly reflects the amount of governmental efficacy.

**Figure 4:**
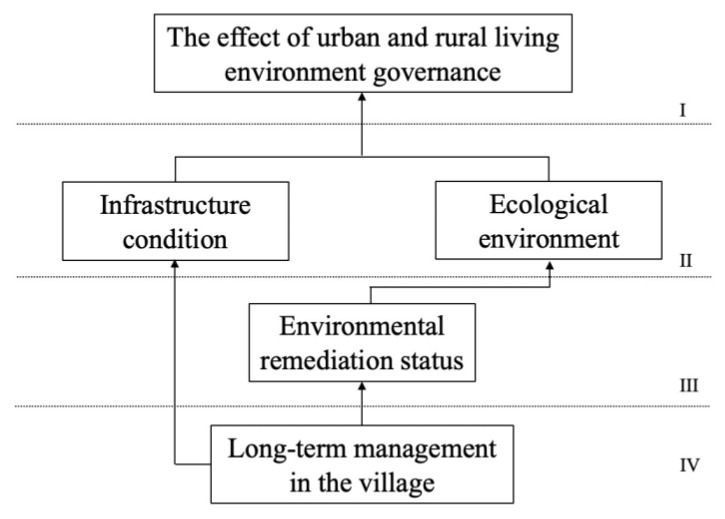
Interpretation of structural model diagram.

The logical relationship between the influencing factors has four layers. The fifth hypothesis has not been validated. The long-term management of the village has a direct impact on the condition of environmental remediation and the state of the infrastructure, whereas environmental remediation has an indirect impact on the ecological environment, ecological environment, and infrastructure capacity. It has a direct impact on how well urban and rural living environments are managed, and the state of environmental improvement has no bearing on infrastructure conditions. This could be due to the fact that residents in the examined villages are more involved in environmental improvement, and infrastructure management is heavily reliant on government finance subsidies. To some extent, the ecological environment has been optimized, making infrastructural improvement less beneficial. The logical relationship between the influencing factors is shown in Figure 4.

Based on the preceding results, the following policy recommendations are provided in this study. The first step in building a long-term management system for the urban–rural environment is to foster collaborative governance among the government, village committees, villagers, businesses, and other topics, as well as to strengthen the multisectoral coordination system. Encourage villagers’ involvement in village issues through expanding public engagement channels, providing guidance, and enhancing multisectoral participation mechanisms. It is critical to establish and improve a long-term mechanism for the comprehensive improvement of the urban rural environment in order to improve the efficiency of governance of the human living environment in the village and achieve sustainable development of the human living environment in urban rural areas [35]. Long-term management must also be integrated into all aspects of environmental and infrastructure improvement, as well as ecological optimization.

Second, we will strengthen policy support for the planning and layout of network cables and wires, improvement of rundown houses, and optimization of environmental improvement measures by developing an incentive system for villagers to participate and improving the villagers’ self-governance mechanism. We will promote the implementation of policy measures such as the restructuring of network connections and wires, as well as the renovation and demolition of derelict homes in rural areas. The vast and dispersed migrant population in urban and rural areas lacks a strong sense of community and belonging. It is critical to increase the village committee’s promotion of collaborative governance, strengthen the villagers’ self-government system, and reduce the cost of governance consultation. To increase the villagers’ sense of worth and accomplishment, as well as to unlock the intrinsic motivation of spiritual incentives, they should be encouraged to participate in environmental improvement through volunteerism and other diverse methods, backed up by an incentive model that includes both material and spiritual rewards.

Third, we coordinate financial investments for long-term environmental upkeep and infrastructure postconstruction management, as well as the integration of ecological and economic elements. We will increase funding for village infrastructure construction and maintenance through the issuance of special bonds and direct government subsidies, attract corporate capital through financial resources and favorable policies, and coordinate and integrate a variety of funding sources, such as village self-funding, social donations, and corporate participation, in order to jointly participate in environmental management, raise farmers’ living standards, and promote organic farming. Third-party organizations are encouraged to participate in the monitoring and assessment of the operation of funds for rural environmental management in order to increase the efficacy of the use of money spent on building.

## 6. Conclusions

The primary purpose of this study was to investigate the villagers’ perspectives on the hurdles to long-term management of urban–rural habitat, as well as the linkages and hierarchical structure between the components. The study employed an ordered logistic-ISM model to investigate the mechanism of long-term urban rural habitat management from the standpoint of villagers, analyze its constraints, and investigate the correlation and hierarchy of the factors. The outcomes demonstrate that the status of environmental improvement, infrastructure conditions, and biological circumstances were discovered to all have a major positive impact on the success of managing urban and rural habitats. This result is consistent with earlier research [13,14,18]. Long-term village management, as a deep-rooted root cause factor, directly contributes to the improvement of the village environment and infrastructure and indirectly contributes to the improvement of the ecological environment through villagers’ participation in environmental initiatives. Long-term village management has a substantial positive impact on the effectiveness of urban rural habitat management, in addition to the state of environmental improvement. While the impact of villager participation in environmental improvement on infrastructure improvement is not immediately obvious, infrastructure improvement and ecological environment optimization as surface-level factors directly promote the success of urban rural habitat management. Because of the more advanced economic growth of urban and rural areas and the government’s strong attention to environmental protection, the impact of factors such as sex and age on the judgment of the efficacy of urban rural habitat management is not immediately obvious. As a result, villagers’ perceptions and assessments of the village environment are more rational and objective. Therefore, by establishing a sound long-term management mechanism for urban rural habitat, the government can effectively improve the village environment, improve infrastructure and ecological environment, and guide villagers to participate in environmental governance, which can increase the endogenous motivation of the government and villagers to participate in urban rural habitat co-management, achieve the maintenance and continuation of the governance effect, and promote sustainable environmental development. Although the findings of this paper can provide some reference for the government’s specific measures and focus on urban rural habitat management and can complement some of the research on urban rural habitat management, the data sample is concentrated in rural Shanghai, and there is a lack of research data from other urban rural areas; the selection of indicators in the evaluation system needs to be further enriched and improved, so we hope that future research will be able to provide more information.

## Figures and Tables

**Table 1:** Basis of selection of indicators.

Indicator Name	Available Literature	Policy Documents	Interviews with Villagers, Village Committees	Construction of this Study
Villagers’ participation	P [13,22]	P	P	P
Supervision and inspection of the environment in the village	P [14]	P	P	P
Villagers’ sense of ownership	P [22]		P	P
Villagers’ willingness to spontaneously protect the rural environment	P [22]		P	P
Promotion of environmental protection in the village	P [13]	P	P	P
Convenience of the distance between public toilets in the village		P	P	P
Convenience of using public toilets in the village	P [2,11,14,18]	P	P	P
Maintenance of public toilets in the village	P [14]	P	P	P
Villagers’ cultural confidence	P [22]	P		P
Village network cable, electric wire condition		P	P	P
The degree of attention paid to the problem of random pulling in the village		P	P	P
Subsidy level for dilapidated and old houses in the village		P	P	P
The completeness of the information published in the village		P	P	P
Villagers’ feelings after demolition of dilapidated houses		P	P	P
Greening facilities in front of and behind the house	P [11]	P	P	P
Water condition in the village	P [11]	P	P	P
Concentrated living conditions in the village		P	P	P
Road conditions in the village	P [2,14]	P	P	P
Water and electricity usage in the village	P [2,11,14]	P	P	P
The convenience of medical treatment in the village	P [11]	P	P	P
Supporting conditions of public activity places in the village	P [2,11]	P		P
Ease of schooling in the village	P [11]	P	P	P
Cultural activities in the village	P [11]		P	P
Supporting conditions of physical exercise venues in the village	P [11]	P		P
Garbage disposal and sanitation in the village	P [2,11,18]	P	P	P

P indicates that the indicator has been selected with reference to this basis.

**Table 2:** Meaning of indicator.

Indicator Name	Assignment and Meaning
Villagers’ participation	Very low = 1; not very high = 2; average = 3; high = 4; very high = 5
Supervision and inspection of the environment in the village	Very little = 1; not too much = 2; average = 3; much = 4; very much = 5
Villagers’ sense of ownership	Very reluctant = 1; Not very willing = 2; Fair = 3; Willing = 4; Very willing = 5
Villagers’ willingness to spontaneously protect the rural environment	Very reluctant = 1; Not very willing = 2; Fair = 3; Willing = 4; Very willing = 5
Promotion of environmental protection in the village	Never = 1; Occasionally = 2; Generally = 3; Often = 4; Very often = 5
Convenience of the distance between public toilets in the village	Very far = 1; relatively far = 2; average = 3; relatively near = 4; very near = 5
Convenience of using public toilets in the village	Very inconvenient = 1; Not very convenient = 2; Fair = 3; Convenient = 4; Very convenient = 5
Maintenance of public toilets in the village	No one cleans = 1; not too much = 2; average = 3; a lot = 4; very much = 5
Villagers’ cultural confidence	Very reluctant = 1; Not very willing = 2; Fair = 3; Willing = 4; Very willing = 5
Village network cable, electric wire condition	Very messy = 1; Messy = 2; Average = 3; Neat = 4; Very neat = 5
The degree of attention paid to the problem of random pulling in the village	No importance = 1; Not much importance = 2; Fair = 3; Important = 4; Very important = 5
Subsidy level for dilapidated and old houses in the village	Very low = 1; not very high = 2; average = 3; high = 4; very high = 5
The completeness of the information published in the village	Very little detail = 1; Not too much detail = 2; Average = 3; Detail = 4; Very much detail = 5
Villagers’ feelings after demolition of dilapidated houses	Little impact = 1; little improvement = 2; fair = 3; much improvement = 4; much improvement = 5
Greening facilities in front of and behind the house	Very unattractive = 1; Not very attractive = 2; Fair = 3; Fairly attractive = 4; Very beautiful = 5
Water condition in the village	Very unclear = 1; Not too clear = 2; Fair = 3; Clear = 4; Very clear = 5
Concentrated living conditions in the village	Little impact = 1; little improvement = 2; fair = 3; much improvement = 4; much improvement = 5
Road conditions in the village	Road very narrow = 1; road relatively narrow = 2; fair = 3; road relatively wide = 4; road very wide = 5
Water and electricity usage in the village	Very inconvenient = 1; Not very convenient = 2; Fair = 3; Convenient = 4; Very convenient = 5
The convenience of medical treatment in the village	Very inconvenient = 1; Not very convenient = 2; Fair = 3; Convenient = 4; Very convenient = 5
Supporting conditions of public activity places in the village	No public space = 1; Yes, almost abandoned = 2; Yes, generally convenient = 3; Yes, relatively convenient = 4; Yes, very convenient = 5
Ease of schooling in the village	Very inconvenient = 1; Not very convenient = 2; Fair = 3; Convenient = 4; Very convenient = 5
Cultural activities in the village	Never held = 1; Occasionally = 2; Generally = 3; Often = 4; Very often = 5
Supporting conditions of physical exercise venues in the village	No place for physical exercise = 1; Yes, almost abandoned = 2; Yes, generally convenient = 3; Yes, relatively convenient = 4; Yes, very convenient = 5
Garbage disposal and sanitation in the village	No waste disposal station = 1; hardly used or abandoned = 2; rarely used and unmanaged = 3; moderately used and unmanaged = 4; well-used and managed = 5

**Table 3:** Factor loading factors after rotation.

Variables	Indicator Name	Factor Loading Coefficient			
		Factor 1	Factor 2	Factor 3	Factor 4
LTM	Villagers’ participation	0.759	-	-	-
	Supervision and inspection of the environment in the village	0.741	-	-	-
	Villagers’ sense of ownership	0.79	-	-	-
	Villagers’ willingness to spontaneously protect the rural environment	0.782	-	-	-
	Promotion of environmental protection in the village	0.769	-	-	-
	Convenience of the distance between public toilets in the village	0.479	-	-	-
	Convenience of using public toilets in the village	0.533	-	-	-
	Maintenance of public toilets in the village	0.567	-	-	-
	Villagers’ cultural confidence	0.637	-	-	-
ERS	Village network cable, electric wire condition	-	0.743	-	-
	The degree of attention paid to the problem of random pulling in the village	-	0.771	-	-
	Subsidy level for dilapidated and old houses in the village	-	0.76	-	-
	The completeness of the information published in the village	-	0.626	-	-
	Villagers’ feelings after demolition of dilapidated houses	-	0.629	-	-
	Greening facilities in front of and behind the house	-	0.471	-	-
	Water condition in the village	-	0.47	-	-
	Concentrated living conditions in the village	-	0.635	-	-
IS	Road conditions in the village	-	-	0.569	-
	Water and electricity usage in the village	-	-	0.556	-
	The convenience of medical treatment in the village	-	-	0.725	-
	Supporting conditions of public activity places in the village	-	-	0.678	-
	Ease of schooling in the village	-	-	0.818	-
	Cultural activities in the village	-	-	0.613	-
	Supporting conditions of physical exercise venues in the village	-	-	0.738	-
EE	Garbage disposal and sanitation in the village	-	-	-	0.906
Cumulative variance contribution rate (%)		23.586	42.857	61.871	67.507

**Table 4:** Control variable description and mean comparison results.

Variable Name	Assignment and Meaning	Mean	Standard Deviation	Z Statistic for Mean Contrast
Age	Age. 1 = under 30 years old, 2 = 31–40 years old, 3 = 41–50 years old, 4 = 51–60 years old, 5 = 60 years old and above	2.271	0.918	-
Edu	Education level. 1 = elementary school and below, 2 = junior high school, 3 = high school or secondary school, 4 = junior college, 5 = undergraduate and above	4.271	0.938	-
Inc	Annual household income. 1 = below 10,000, 2 = 1–30,000, 3 = 3–50,000, 4 = 50,000–100,000, 5 = more than 100,000	3.929	1.285	-
Sex	Sex. 0 = woman, 1 = man	0.414	0.493	−0.665
Lead	Whether the village cadre. 0 = no, 1 = yes	0.368	0.483	−4.699 ***
Rrdv	Whether it is a rural revitalization model village. 0 = no, 1 = yes	0.279	0.449	−2.502 **
Cca	Concentrated construction area. 0 = nonconcentrated construction area, 1 = concentrated construction area	0.275	0.447	−1.785 *

***, **, and * indicate significance at the 1%, 5%, and 10% levels, respectively, the same below.

**Table 5:** Regression results of ordered logistic model.

Explanatory Variables	Model 1	Model 2
	Regression Coefficients (Standard Error)	Regression Coefficients (Standard Error)
EE	0.616 *** (0.140)	0.569 *** (0.136)
IS	1.014 *** (0.160)	1.030 *** (0.157)
ERS	1.093 *** (0.176)	1.081 *** (0.169)
LTM	1.660 *** (0.192)	1.614 *** (0.183)
Sex	−0.014(0.317)	-
Age	−0.104(0.184)	-
Edu	−0.039(0.191)	-
Inc	−0.096(0.140)	-
Lead	0.647 * (0.372)	-
Rrdv	−0.261(0.404)	-
Cca	0.514(0.392)	-
Likelihood ratio test	210.536 ***	207.482 ***
McFadden R^2^	0.402	0.396

*** and * indicate significance at the 1% and 10% levels, respectively.

**Table 6:** Robustness test results.

Variable	Model 3		Model 4	
	Cca = 0 (Nonconcentrated Construction Area)	Cca = 1 (Concentrated Construction Area)	Rrdv = 0 (Nonrural Revitalization Demonstration Village)	Rrdv = 1 (Rural Revitalization Demonstration Village)
	Regression Coefficients (Standard Error)	Regression Coefficients (Standard Error)	Regression Coefficients (Standard Error)	Regression Coefficients (Standard Error)
Constant	4.481 *** (125.097)	4.593 *** (85.565)	4.510 *** (117.211)	4.489 *** (98.239)
LTM	0.457 *** (13.470)	0.366 *** (5.899)	0.447 *** (12.291)	0.378 *** (7.176)
ERS	0.244 *** (6.687)	0.184 *** (3.616)	0.224 *** (6.195)	0.286 *** (4.885)
IS	0.266 *** (7.524)	0.168 *** (2.965)	0.259 *** (7.045)	0.212 *** (4.408)
EE	0.199 *** (5.343)	0.126 ** (2.608)	0.163 *** (4.669)	0.246 *** (3.874)
N	203	77	202	78
R²	0.614	0.47	0.562	0.647
Adjusted R²	0.606	0.441	0.553	0.627

*** and ** indicate significance at the 1% and 5% levels, respectively.

## Data Availability

The data in this paper come from a survey conducted in 2021 in nine farming-related districts in Shanghai, and field research was conducted in a number of villages and towns in farming-related districts in Shanghai.

## Appendix A

The appendix shows the survey questionnaire on villagers’ satisfaction with the rural habitat in Shanghai.

Hello! We are researchers at the Shanghai Ocean University Branch of the Shanghai Social Research Centre and are conducting a survey on villagers’ satisfaction with the rural habitat in Shanghai, with the aim of finding out how satisfied they are with the rural habitat in Shanghai and providing suggestions for better development and construction of the rural habitat in Shanghai. This questionnaire is for academic research purposes only and is conducted anonymously, with all relevant information kept confidential. Your support and participation are greatly appreciated!

Your gender is [Single choice]

○Male ○Female

Your age is [Single choice]

○Under 30 years old ○31–40 years old ○41–50 years old ○51–60 years old ○60 years old or above

Your level of education is [Single choice]

○Elementary school and below ○Junior high school ○High school/junior high school ○College ○Bachelor’s degree and above

What is your total annual household income (yuan)? [Single choice]

○Less than 10,000 ○10,000–30,000 ○30,000–50,000 ○50,000–100,000 ○Over 100,000

Are you a village official? [Single choice]

○Yes ○No

Is your village a model village for rural revitalization? [Single choice]

○Yes ○No

Is your village part of a catchment area? [Single choice]

○Yes ○No

Which agricultural zone do you belong to? [Single choice]

○Pudong New District ○Chongming District ○Jinshan District ○Fengxian District ○Qingpu District ○Songjiang District ○Jiading District ○Baoshan District ○Minhang District

1. How satisfied are you with the environment of your village? [Single choice]

○Very satisfied ○Satisfied ○Fair ○Not very satisfied ○Very dissatisfied

2. Do you currently have a high level of participation in the construction of beautiful villages? [Single choice]

○Very high ○High ○Fair ○Not too high ○Very low

3. How often do you think government staff monitor and inspect the environment on a daily basis? [Single choice]

○Very often ○Much ○Fairly ○Not too much ○Very little

4. If you see other villagers committing violations, are you willing to take the initiative to stop them? [Single choice]

○Very willing ○Willingly ○Generally ○Not very willing ○Very reluctant

5. Are you willing to take the initiative to pick up litter when you see it in the village? [Single choice]

○Very willing ○Willing ○Generally ○Not very willing ○Very reluctant

6. Does the village council often hold environmental protection publicity activities? [Single choice]

○Very often ○Frequently ○Very often ○Sometimes ○Never

7. Which of the following measures do you think will improve environmental management in the village? [Multiple choice](Limit 1–3 items)

○ Enhance the supervision of supervisors

○ Raise the awareness of villagers to participate in environmental management in the village

○ Increase government funding

○ Enhance the operability of government inspection indicators

○ Others _________________

8. How far do you think the public toilets in your village are from your home? [Single choice]

○Very close ○Rather close ○Fair ○Far away ○Very far away

9. Do you think it is convenient to use public toilets after the reform of toilets in the village? [Single choice]

○Very convenient ○Convenient ○Ordinary ○Not very convenient ○Very inconvenient

10. How often do the staff clean and maintain the village toilets on a daily basis? [Single choice]

○Very often ○Much ○Average ○Not too much ○No one cleans

11. Which of the following measures do you think would improve the condition of public toilets in the village? [Multiple choice](Limit 1–3 items)

○ The government to increase the implementation of construction land quota for the construction of public toilets

○ Improve toilet infrastructure facilities and increase the number of public toilets

○ Raise villagers’ awareness of the need to regulate the use of toilets

○ Shorten the distance between dwellings and public toilets

○ Increase government funds for management and maintenance

○ Install more mobile public toilets

○ Other _________________

12. What is the method of domestic sewage treatment used in your own home? [Single choice]

○The village collects sewage through a unified sewerage pipe and builds treatment facilities to treat it

○You build your own septic tank and other facilities for treatment

○Other _________________

13. Are you satisfied with the way domestic sewage is treated in your village? [Single choice]

○Very satisfied ○Satisfied ○Fair ○Not very satisfied ○Very dissatisfied

14. Do you think the arrangement of the network cables and wires in the village air is messy? [Single choice]

○Very neat ○Tidy ○Ordinary ○Messy ○Very messy

15. Do you think the government attaches importance to the problem of indiscriminate pulling of wires and cables in the air? [Single choice]

○Very important ○Important ○Generally ○Not very important ○Never attached importance

16. Which of the following measures do you think can improve the situation of indiscriminate pulling of Internet cables and wires in the village? [Multiple choice](Limit 1-3 items)

○ Strengthen the coordination and construction role of the Information and Communication Development Department of the State Ministry of Industry and Information Technology

○ Improve the level of cooperation and coordination between telecom operators, power supply bureaus and village committees

○ Uniform layout planning of village network lines and power lines to enhance the rationality of planning

○ Decentralize households and reduce the degree of density

○ Other _________________

17. To what extent do you think the government subsidizes the renovation and demolition of dilapidated houses? [Single choice]

○Very high ○High ○Fair ○Not too high ○Very low

18. When the notice of house renovation and demolition is posted in the village, is the information on the notice detailed? [Single choice]

○Very detailed ○Detailed ○Generally ○Not very detailed ○Very little detail

19. Do you think the demolition and renovation of houses has improved the environment in the village? [Single choice]

○Much improved ○More improved ○Fairly ○Less improved ○Not much impact

20. Which of the following measures do you think will improve the condition of renovation and demolition of dilapidated houses in the village? [Multiple choice](Limit 1-3 items)

○ Increasing government subsidies

○ Increasing the degree of unity of opinion among household heads

○ Increase the degree of villagers’ concern about dilapidated houses

○ Enhance the degree of cooperation of village committees

○ Others _________________

21. What do you think of the greenery in front and behind your home? [Single choice]

○Very beautiful ○Rather beautiful ○Fair ○Not very beautiful ○Very unattractive

22. Is there a designated rubbish disposal station in the village? If yes, how is it used and managed? [Single choice]

○They are well used and managed ○Use is fair and no one manages it ○ Rarely used and not managed ○Never used or abandoned ○No rubbish disposal station

23. Do you think the water in the village’s rivers is clear? [Single choice]

○Very clear ○Clear ○Fair ○Not very clear ○Very unclear

24. What is the centralized settlement used in your village? [Single choice]

○Flat ○Upstairs ○None ○Other _________________

25. Do you think that the environment of the village can be improved after the village is centrally housed (upstairs or levelling)? [Single choice]

○Improved a lot ○Improved a lot ○Fairly ○Less improvement ○Not much impact

26. What are the main problems with the centralization of villagers’ housing? [Multiple choice](Limit 1–3 items)

○ Poor government subsidies

○ Disputes over property rights among villagers

○ Low willingness of villagers to live centrally, longing for the original rural life

○ Concentrated housing and beautification of the habitat of the original village are carried out at the same time, resulting in duplication and waste of construction resources, and unified planning is not done in advance

○ Incomplete relocation of centralized housing, e.g., residential bases and self-reserved land are not handled in a unified manner

○ Other _________________

27. What do you think of the condition of the roads in the village? [Single choice]

○The road surface is very wide ○The road surface is relatively wide ○Fair ○The road surface is narrow ○The road surface is very narrow

28. How convenient do you think it is to use water and electricity in the village? [Single choice]

○Very convenient ○Convenient ○Fair ○Not very convenient ○Very inconvenient

29. Do you think it is convenient (in terms of distance from home) to go to the hospital within the village? [Single choice]

○Very convenient ○Convenient ○Fair ○Not very convenient ○Very inconvenient

30. Are there any public places in the village, such as small parks and chess and card rooms? If yes, is it convenient to use? [Single choice]

○Yes, very convenient ○Yes, quite convenient ○Yes, generally convenient ○Yes, almost abandoned ○No public activity space

31. How convenient do you think it is for your children to go to school at home (in terms of the distance of the school from home)? [Single choice]

○Very convenient ○Convenient ○Fair ○Not very convenient ○Very inconvenient

32. How often do you think the festivals are held in your village? [Single choice]

○Very frequently ○Frequently ○Very often ○Sometimes ○Never held

33. Is there a place in the village that provides sports and exercise? If so, is it easy to use? [Single choice]

○Yes, very convenient ○Yes, quite convenient ○Yes, generally convenient ○Yes, almost abandoned ○No place for physical exercise

34. Would you be willing to promote the traditional culture of your village to others? [Single choice]

○Very willing ○Willing ○Generally ○Not very willing ○Very reluctant
